# IFNα and IFNγ Impede Marek’s Disease Progression

**DOI:** 10.3390/v11121103

**Published:** 2019-11-28

**Authors:** Luca D. Bertzbach, Olof Harlin, Sonja Härtle, Frank Fehler, Tereza Vychodil, Benedikt B. Kaufer, Bernd Kaspers

**Affiliations:** 1 Institute of Virology, Freie Universität Berlin, 14163 Berlin, Germany; luca.bertzbach@fu-berlin.de (L.D.B.); tereza.vychodil@fu-berlin.de (T.V.); 2 Department of Veterinary Sciences, Ludwig-Maximilians-Universität München, 80539 Munich, Germany; oharlin@yahoo.com (O.H.); sonja.haertle@lmu.de (S.H.); 3 Lohmann Animal Health, 27472 Cuxhaven, Germany; frank.fehler@web.de

**Keywords:** Marek’s disease virus, recombinant interferons, chicken cytokines, innate immunity, antiviral host defense, antitumor immune response

## Abstract

Marek’s disease virus (MDV) is an alphaherpesvirus that causes Marek’s disease, a malignant lymphoproliferative disease of domestic chickens. While MDV vaccines protect animals from clinical disease, they do not provide sterilizing immunity and allow field strains to circulate and evolve in vaccinated flocks. Therefore, there is a need for improved vaccines and for a better understanding of innate and adaptive immune responses against MDV infections. Interferons (IFNs) play important roles in the innate immune defenses against viruses and induce upregulation of a cellular antiviral state. In this report, we quantified the potent antiviral effect of IFNα and IFNγ against MDV infections in vitro. Moreover, we demonstrate that both cytokines can delay Marek’s disease onset and progression in vivo. Additionally, blocking of endogenous IFNα using a specific monoclonal antibody, in turn, accelerated disease. In summary, our data reveal the effects of IFNα and IFNγ on MDV infection and improve our understanding of innate immune responses against this oncogenic virus.

## 1. Introduction

The highly oncogenic Marek’s disease virus (MDV) infects chickens and is a major burden for poultry farming worldwide. Despite the widespread use of vaccines, MDV remains a serious threat to poultry and causes substantial economic losses worldwide every year [1]. This lymphotropic alphaherpesvirus replicates in different immune cell types such as B and T cells [2] and can establish a latent infection of CD4+ T cells which is a prerequisite for malignant transformation of these cells [3,4]. Clinical signs of Marek’s disease in chickens include torticollis, ataxia, and paralysis of the legs and wings due to an enlargement of peripheral nerves [5]. In susceptible birds, MDV infection results in the formation of deadly T cell lymphomas in up to 100% of the animals [3]. 

Interferons (IFNs) are cytokines that possess strong antiviral properties and are a major component of the innate antiviral host defense. They can be divided into type I (IFNα and IFNβ), type II (IFNγ), and type III (IFNλ) IFNs, based on their structural and functional features [6]. While type I IFNs are secreted by many different cell types, IFNγ is predominantly produced by T helper 1 cells and natural killer cells [6]. The roles of IFNλ remain poorly understood [7,8]. It has been shown that IFNs are expressed as an antiviral response to MDV infections in vitro [9,10] and in infected chickens [11,12,13,14,15,16]. Interestingly, a study by Jarosinski et al. describes that oral administration of IFNα reduces MDV replication in experimentally infected chickens, while no data on disease onset and progression are available [17]. The major aim of this study was to assess the effect of IFNs on MDV replication properties in vitro and to determine if recombinant chicken IFNα and IFNγ could impair disease onset and progression in chickens and thereby further elucidate the roles of these cytokines in MDV infections.

With this report, we could demonstrate that (i) IFNα and IFNγ inhibit MDV in vitro replication in a dose-dependent manner, that (ii) the antiviral response of primary chicken fibroblasts on MDV infection is IFNα-mediated, and that (iii) IFNα and IFNγ significantly impair disease progression in infected animals. 

## 2. Materials and Methods

### 2.1. Cells and Viruses

Chicken embryo cells (CEC) were prepared from specific-pathogen-free (SPF) chicken embryos (Valo BioMedia; Osterholz-Scharmbeck, Germany) that were incubated in-house. CEC were cultured in minimum essential medium (MEM, PAN Biotech; Aidenbach, Germany) supplemented with 1–10% fetal calf serum (FCS; PAN Biotech) and 1% antibiotics (100 U/mL penicillin and 100 µg/mL streptomycin; AppliChem; Darmstadt, Germany) at 37 °C in a humidified atmosphere containing 5% CO_2_ [18]. B cells were obtained as previously described and cultured in Iscove’s basal medium with 8% FCS, 2% chicken serum, and penicillin/streptomycin and activated with recombinant chCD40L [19,20,21]. For in vitro assays, we used the very virulent RB-1B strain [22] and the vaccine strain CVI988 [23]. The very virulent plus Italian MDV-1 strain EU-1 [24] was used in both animal experiments.

### 2.2. Chicken Interferons and Antibodies

Recombinant chicken IFNα (rChIFNα) and recombinant chicken IFNγ (rChIFNγ) were produced in *Escherichia coli* [25,26] and protein concentrations were determined using the Bradford assay [25,27]. Polyclonal rabbit anti-IFNα and polyclonal rabbit anti-IFNβ were obtained as previously described [25,28] and the monoclonal antibody (mAb 8A9) against chicken IFNα was obtained from a rat as reported earlier [29].

### 2.3. In Vitro Assays

Replication properties and cell-to-cell spread of the MDV strain RB-1B in CEC were determined by plaque size assays in the presence or absence of the IFNs in three independent biological replicates as previously described [30]. MDV infection in primary chicken B cells was assessed by flow cytometry as previously described [20]. B cells were infected with RB-1B by co-cultivation with infected CEC in the presence and absence of IFNα. To test the antiviral activity of MDV-infected cell culture supernatants, we used the vesicular stomatitis Indiana virus (VSV) bioassay as described by Lewis [31,32,33] (Figure S1). Briefly, VSV replication is highly susceptible to IFNs, a property used to assess the presence of IFNs in cell culture supernatants. Supernatants of MDV-infected CEC were added to a monolayer of CEC-32 cells and infected with VSV. Cells were stained with neutral red, washed with phosphate-buffered saline (PBS), lysed, and the optical density measured at 540 nm. The amounts of viable cells protected by IFNs corresponds to the optical density [31,33]. To confirm the specificity, the antiviral activity could be blocked by addition of anti-IFNα and anti-IFNβ antisera or an anti-IFNα mAb (Figure S1).

### 2.4. Ethics Statement and Animal Experiments

This report describes two animal studies. In both, SPF Lohmann selected leghorn (LSL; Lohmann Animal Health, Cuxhaven, Germany) were hatched and kept under SPF conditions. The studies were approved by the responsible authority (the Animal Research Board of the State of Lower Saxony, Germany; animal use protocol # 295/01 (IFNα) and 45/01 (IFNγ)) and were conducted according to relevant national and international guidelines for the humane use of animals. Chickens had ad libitum access to food and water, and were routinely checked for clinical signs like ataxia, ruffled feathers, and somnolence throughout the 70 day experiments. 

In the first animal study, 15 chickens per treatment group (1: mock/PBS, 2: IFNα (250 IU), 3: anti-IFNα mAb (100 µg)) were injected intramuscularly with a low dose of the very virulent + (vv+) strain EU-1 at 2 days post-hatching (100 µL lymphocyte suspension). Treatments with IFNα and anti-IFNα mAb were administered intraperitoneally: The first treatment was given at 1 day post-hatching and all chickens received repeated treatment every third day over the period of 10 weeks.

In the second animal experiment, seven chickens per group (1: mock/PBS, 2: IFNγ (200 IU)) were intramuscularly infected with a high dose of the vv+ strain EU-1 (200 µL lymphocyte suspension) at 2 days post-hatching. Treatments were administered intraperitoneally: The first treatment was given at 1 day post-hatching and all chickens received repeated treatment every third day. 

The successful establishment of infection was confirmed in blood samples of three animals per group by PCR detecting the infected cell protein 4 (*ICP4*) gene of MDV (Tables S1 and S2) [34]. All chickens were humanely euthanized and examined post-mortem for tumor lesions once clinical symptoms appeared or upon the termination of the experiment. Tumors were mainly detected in the visceral organs (spleen, liver, and kidneys).

### 2.5. Statistical Analyses

Statistical analyses were performed with Graph-Pad Prism v5. One-way analysis of variance (ANOVA) with Bonferroni correction on multiple comparisons was used for plaque size assays and the effect of IFN on the infection of primary chicken B cells. Kaplan–Meier survival analysis along with the Mantel–Cox test (log-rank test) was used for analyses of the animal experiment data. Data were considered significantly different if *p* ≤ 0.05.

## 3. Results and Discussion

### 3.1. Effect of IFNα on MDV Replication and Pathogenesis

**Figure 1:**
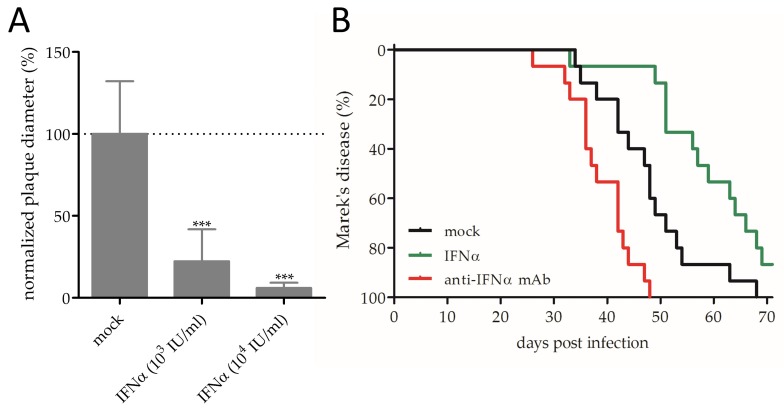
Anti-Marek’s disease virus (MDV) effects of interferon-alpha (IFNα): (**A**) Dose-dependent replication inhibition upon IFNα treatment, as assessed by conventional plaque size assays (*** *p* < 0.001, one-way ANOVA with Bonferroni correction). (**B**) Kaplan–Meier analysis of Marek’s disease incidence in chickens with indicated treatment (Mantel–Cox test; *p* < 0.001).

To assess the effect of recombinant IFNα on MDV replication in vitro, we performed plaque size assays and observed a dose-dependent effect on virus replication in CEC (Figure 1A) and in primary chicken B cells (Figure S2). This is consistent with previously described reduction in the plaque numbers upon IFNα treatment of cells [17,35]. 

Moreover, we could demonstrate that IFNα, but not IFNβ, was released into the supernatant upon infection of primary CEC with MDV (Figure S1). In line with this, it has recently been shown that the MDV-encoded protein RLORF4 inhibits IFNβ production in chicken fibroblasts [36]. These data confirm the IFNα-mediated antiviral response on the protein level—a response that has so far only been shown on the RNA level [9]. In experimentally infected chickens that were treated with IFNα, treatment significantly delayed the disease incidence with median survival rates of mock and IFNα treated animals of 48 and 59 days respectively (Figure 1B; *p* < 0.05). These findings indicate that administration of IFNα has a protective effect, but that it cannot prevent disease in MDV-infected chickens. This could be due to the lower efficacy of IFNα in inhibiting MDV infection in primary chicken B cells (Figure S2). In those cells, high concentrations of IFNα only reduced MDV infection to 54% and 49% (±12.1 standard deviation) 24 and 48 hours post-infection, respectively, indicating that the potency of IFNα to suppress MDV replication substantially differs in different cell types.

Furthermore, we could demonstrate that treatment with anti-IFNα mAb accelerates the onset of disease, highlighting the important role of early IFNα responses. The median survival was reduced to 38 days and it took approximately 3 weeks less until all animals showed clinical signs of Marek’s disease (Figure 1B; *p* < 0.05). Taken together, we could demonstrate that IFNα impairs MDV replication in vitro and that it extends the survival of treated chickens. Moreover, the important role of IFNα in MDV infection was confirmed by blocking endogenous IFNα with mAb.

### 3.2. Effect of IFNγ on MDV Replication and Pathogenesis 

**Figure 2:**
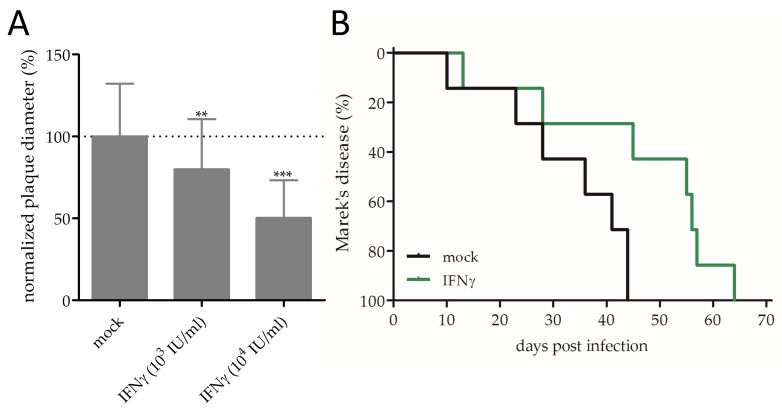
Anti-MDV effects of interferon-gamma (IFNγ): (**A**) Dose-dependent replication inhibition upon IFNγ treatment, as assessed by conventional plaque size assays (** *p* < 0.01 and *** *p* < 0.001, one-way ANOVA with Bonferroni correction). (**B**) Kaplan–Meier analysis of Marek’s disease incidence in chickens with indicated treatment (Mantel–Cox test; *p* = 0.0226).

Similar to IFNα, we also observed a dose-dependent inhibition of MDV replication upon IFNγ treatment in vitro (Figure 2A). This data is consistent with previously described IFNγ-dependent reduction in the plaque numbers for different MDV strains and turkey herpesvirus [37]. Interestingly, the effect of treatment was less pronounced when compared to IFNα treatment. Both IFNs are known to shape the initial inflammatory and downstream adaptive immune responses. In addition, it is likely that direct antiviral effects of IFNγ are broader and less specific due to its role in the upregulation of inducible nitric oxide synthase expression [38] and other immunomodulatory functions. IFNα, on the other hand, is known to be one of the first cytokines produced during a virus infection and induces an immediate induction of antiviral interferon-stimulated genes. 

Notably, treatment of experimentally infected chickens with IFNγ also led to a delay of disease progression (Figure 2B; *p* < 0.05). Here, we observed a median survival of 36 days (mock) and 55 days (IFNγ). It has been shown that MDV tumor tissue contains elevated levels of interferon-induced proteins, amongst them IFNγ-inducible protein 30 (IFI30), which has been discussed to possess antitumor properties [39,40]. Hence, IFNγ could not only inhibit MDV replication, but also induce an antitumor response [41]. The observed differences in the median survival rates of the mock groups in the IFNα and IFNγ in vivo experiments could be explained by the different doses of MDV infections (see Section 2.4).

## 4. Conclusions

Collectively, these data show that interferons efficiently inhibit MDV replication and significantly delay Marek’s disease progression. Nevertheless, treatment with IFNα and IFNγ alone did not prevent disease and tumor formation in our studies. It remains unclear how MDV circumvents IFN-mediated host immune responses and how this oncogenic alphaherpesvirus could alter IFN production to successfully infect, replicate, shed, and cause clinical disease. The observed discrepancy between in vitro and in vivo results could be explained by the observed differences in the efficacy of IFN treatment on MDV replication in different cell types in vitro with less pronounced antiviral activity in B cells, the primary target cells for MDV replication. Future research could focus on synergistic effects of IFNα and IFNγ [42] or the use of recombinant chicken IFNs in vaccine formulations to boost the chicken immune system to ward off viral diseases [43,44,45]. Beyond type I and II IFNs, the role of type III IFNs should be investigated in the context of MDV infection at mucosal surfaces. Finally, novel methodology in avian immunology and infectious diseases research such as genetically modified chickens [46] or the increasing availability of avian cell markers as well as in vitro and in vivo tools [47] will certainly improve our understanding of these processes in the near future.

## Supplementary Materials

The following are available online at https://www.mdpi.com/1999-4915/11/12/1103/s1, Figure S1: Antiviral activity of chicken embryo cells (CEC) culture supernatant; Figure S2: IFNα-mediated MDV replication inhibition in primary chicken B cells; Table S1: Primers used for the ICP4 PCR; Table S2: PCR confirmation of MDV infections in animals.
