# Supplementary material for: IFNα and IFNγ Impede Marek’s Disease Progression
Source: Viruses. 2019 Nov 28;11(12):1103. doi: 10.3390/v11121103 (PMC6950089; doi:10.3390/v11121103)
Supplement: Supplementary file 1 [file viruses-11-01103-s001.pdf]

viruses

MDPI

Supplementary Material to "IFNα and IFNγ Impede Marek's Disease Progression" by Bertzbach LD et al. (2019)

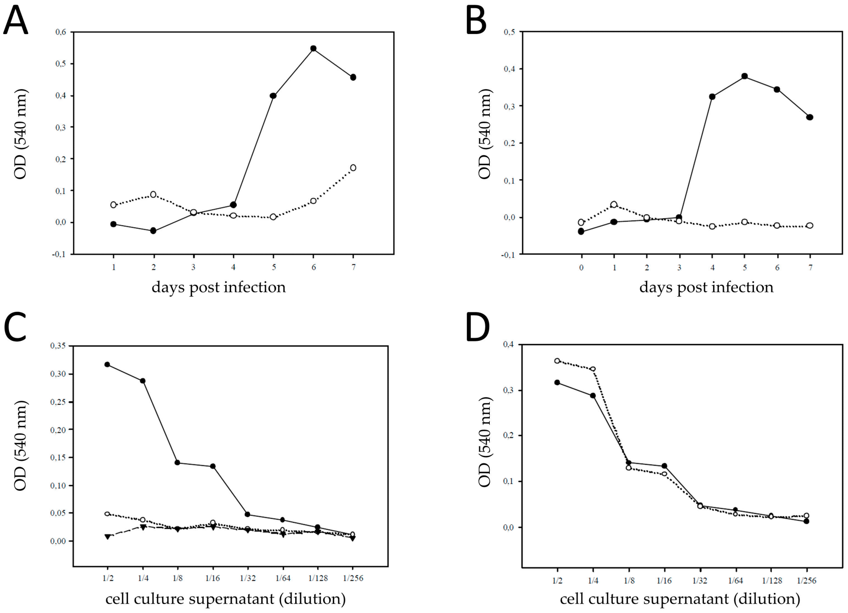

Figure S1: Antiviral activity of chicken embryo cells (CEC) culture supernatant. Measurements of antiviral activity from CVI-infected (**A**) and RB-1B-infected (**B**) CEC. Solid lines indicate infected and dotted lines mock-infected cell culture supernatant (CCS). Optical density (OD) values correlate with antiviral activity. Neutralization of antiviral activity in CCS from RB-1B infected cells specific for IFNα (**C**) and IFNβ (**D**). The solid lines indicate CCS from infected cells and dotted lines are CCS blocked with an anti-IFNα monoclonal antibody (**C**). Dashed lines are CCS blocked with anti-IFNα antiserum (1:80, **C**) or with anti-IFNβ antiserum (1:80, **D**).

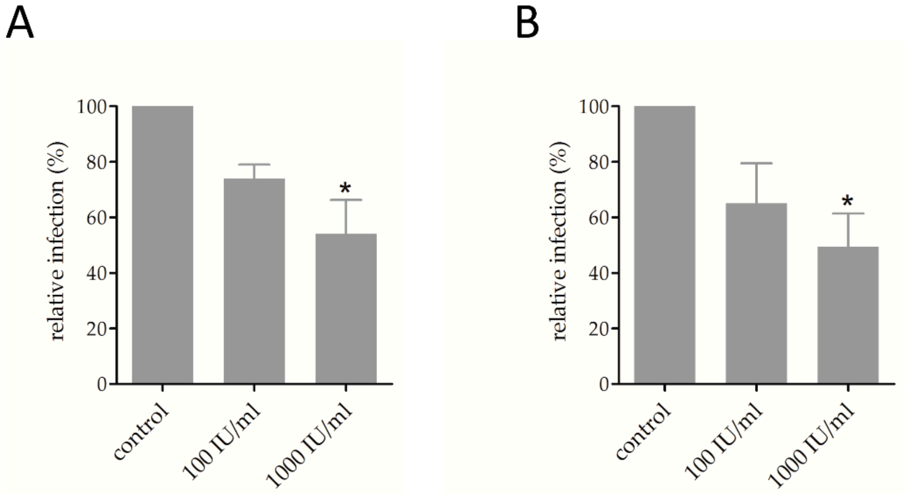

Figure S2: IFNα-mediated inhibition of MDV replication in primary chicken B cells. To assess the replication properties of MDV in primary chicken B cells, cells were infected by co-cultivation and analyzed by FACS. (**A**) Effect of IFNα treatment on MDV replication at 24 hours post infection (h.p.i.) with indicated concentrations, normalized to the untreated control. (**B**) Same analysis at 48 h.p.i. Asterisks indicate significant differences to mock (one-way ANOVA, $^{*}p < 0.05$, $n = 3$). Error bars represent standard deviations.

Table S1. Primers used for the ICP4 PCR.

Primers	Sequence (5' → 3')
ICP4 forward primer	AATGAGCGAACTGCCTCACACAAC
ICP4 reverse primer	GATCGCCCACCACGATTACTACCT

Table S2. PCR confirmation of MDV infections in animals.

	**Group**	**ICP4 PCR positive**
**Experiment 1**	mock	3/3
	IFNα	3/3
	anti-IFNα mAB	3/3
**Experiment 2**	mock	3/3
	IFNγ	3/3
